# Development, Validation, and Evaluation of Web-Based Iranian Diabetic Personal Health Record: Rationale for and Protocol of a Randomized Controlled Trial

**DOI:** 10.2196/resprot.5201

**Published:** 2016-03-10

**Authors:** Amirabbas Azizi, Robab Aboutorabi, Zahra Mazloum-Khorasani, Monavar Afzal-Aghaea, Mahmood Tara

**Affiliations:** ^1^ Faculty of Medicine Department of Medical Informatics Mashhad University of Medical Sciences Mashhad Islamic Republic Of Iran; ^2^ School of Allied Health Professions Department of Health Information Technology Ahvaz Jundishapur University of Medical Sciences Ahvaz Islamic Republic Of Iran; ^3^ Endocrine Research Center Mashhad University of Medical Sciences Mashhad Islamic Republic Of Iran; ^4^ School of Health Health Sciences Research Center Mashhad University of Medical Sciences Mashhad Islamic Republic Of Iran

**Keywords:** diabetes mellitus, type 2, personal health record, web-based, Iran

## Abstract

**Background:**

Diabetes is one of the four main types of noncommunicable or chronic diseases. Iran is among the countries with the highest incidence of diabetic patients. A study demonstrated that the collection of diabetic data is neither organized nor standardized. There is currently no instance of electronic personal health records particularly used for diabetic patients in Iran, hence the need for one, which will be useful for self-care of diabetic patients.

**Objective:**

The objective of the study is to examine the impact of a Web-based diabetic personal health record (DPHR) on the self-care status of diabetic patients as compared with the control group.

**Methods:**

This study is a randomized control trial, which involves a systematic review of literature of the preferred data elements regarding a DPHR, and reevaluating the results with the opinions of local endocrinologists. Inclusion criteria were as follows: type 2 diabetic patients between 20-70 of age who live in the Mashhad City and having the disease for at least one year. The sample size is 72 people that were randomly assigned to the control and intervention groups. The participants in the intervention group were allowed access to the Web-based DPHR system, while those in the control group will continue to receive the usual care for 4 months. The study primary outcome measures include self-care status of participants and planned visit adherence.

**Results:**

At the moment, there is an ongoing recruitment of participants, and preliminary results will be published in early 2016.

**Conclusions:**

We expect the final DPHR model, developed and tested during this study, to help diabetic patients to actively participate in their care management process, and also to empower the physician in providing more quality informed decisions regarding their patients.

**Trial Registration:**

irct.ir IRCT2013082914522N1; http://www.irct.ir/searchresult.php?id=14522&number=1 (Archived by WebCite at http://www.webcitation.org/6cC4PCcau).

## Introduction

### Diabetes Definition

Diabetes is one of the four main types of chronic or noncommunicable diseases [1], and its management is of great concern to the society and world at large [2-5]. A definition by the World Health Organization [6] states that: “Diabetes is a chronic disease that occurs either from insufficient production of insulin by the pancreas or ineffective use of produced insulin”. Type 2 diabetes results from the body’s ineffective use of insulin.

Lack of or insufficient care for a diabetic patient can result in several complications such as diabetic neuropathy, retinopathy, nephropathy, diabetic foot, and myocardial infarction [2]. However, continuous blood glucose and blood pressure monitoring, timely visits, appropriate physical activity, blood lipids control, and periodical examinations, as foundation of diabetes self-management, can help patients lessen these complications [7-9].

In recent times, there has been increasing prevalence of diabetes globally, with the low- and middle-income countries having the highest prevalence. The global prevalence of diabetes was estimated to be 9% in 2014 [10], with an estimated increase of 366,000,000 worldwide and 6,421,000 in Iran by 2030 [11,12]. In order to manage the increasing number of diabetics in the future [13,14], and to reduce the workload of health care providers, there is a need to redefine the role of diabetes management centers [15]. Patients who have more knowledge about their disease and procedures are more proficient at communicating experiences and then become a useful asset in long-term care [16]. In this way, Web-based personal health records (PHRs) provide patients with access to their health information [17-19].

### Personal Health Records

Application of PHRs, in general, and diabetes-specific PHRs, has been growing very rapidly. Studies have demonstrated that Web-based diabetes management tools can improve self-care activities [19] and biomedical outcomes measures such as glycated hemoglobin (HbA1c), low-density lipoprotein (LDL) cholesterol, blood pressure, and body mass index (BMI) [20-24]. The benefits of PHRs in supporting self-management is clear, especially in facilitating communications among health care providers and supporting information access [25].

Iran is among the countries with the highest incidence of diabetic patients [26]. A recent study demonstrated that the collection of diabetic data are neither organized nor standardized [27]. There are no diabetic PHRs (DPHRs) used to organize diabetes data in the country.

This multiphase study involves: (1) a systematic review of literature regarding the preferred data elements regarding a DPHR, (2) an afterward refinement of data elements by the local endocrinologists, (3) a systemic development of a Web-based DPHR application (app), and (4) final evaluation of the model through a randomized controlled trial. The researchers hope that the final model developed throughout in this study will help diabetes patients to actively participate in their treatment plan, and also optimize the decision making process of the diabetes physicians in their everyday practice.

### Study Hypothesis

The participants assigned to receive the DPHR tool will manage better self-care as compared to those who receive only usual care.

## Methods

### Randomization and Blinding

After the provision of informed consent, participants are randomly allocated to the control or intervention arm stratified by gender (male, female), employment status (employed and nonemployed), and age groups (=<30; 30-50; and =>50 years of age), that is, the covariate adaptive random allocation is considered. The random allocation sequence will be done through a concealed and computerized random number program [28]. A person who has no direct involvement in this trial will do the randomization process. Also, we note that the participants and attending physician (RA) can’t be blinded to the use of PHRs because the artefact is obvious, but the chief researcher (MT) and data analyst (MAA) are blinded to the participants during the trial. To minimize contamination of the trial, the subjects in the intervention arm will be required not to interact and share their DPHRs information with other participants in the control arm.

### Study Design

A four-phase approach, including a systematic review of evidence regarding DPHR development, usability testing, iterative refinement, and intervention evaluation will be utilized. A randomized controlled trial (RCT) with a 2-arm parallel design and allocation ratio of 1:1 will be used to evaluate the impact of DPHRs on self-care. Repeated assessments will be conducted at two time points, including baseline and postintervention, over a 4-month trial period. The patients in the intervention group will be allowed access to the Web-based app, while those in the control group will be receiving usual care throughout the study period.

The DPHR interface will be designed in three types, covering our three groups of users including the participating patients, the clinic staff, and the physicians. The chief researcher will be the system administrator. Reminders via a short message service (SMS), telephone contact, and email will be used for visiting the website.

### Trial Population and Recruitment Procedure

The inclusion criteria for participants are as follows: the patient must be a male or female with type 2 diabetes, age between 20 and 70 years, with a minimum diabetes history of one year (according to the primary diagnosis date), must live in the city of Mashhad, must be fairly computer literate, and have access to the Internet. The included participants must be able to provide informed consent.

We excluded the already included participants, if they, for any reason, decided to terminate the participation, or were unable to actively continue the required cooperation, due to sickness, pregnancy, and etc.

The trial will be conducted in Mashhad City (Iran), with participants being recruited from one endocrinology practice unit. The number of diabetic patients is estimated to be above 120,000 in Mashhad [29]. We will start the study with the acquisition of baseline information including age, education level, gender, self-care score, HbA1c, weight, employment status, length of disease, and diagnosis date along with the information required to check the inclusion eligibility. Only the qualified participants will be given the consent form.

After signing the consent form, participants will be randomized into either the intervention or the control arm. After providing confirmed consent, a package including a copy of the consent form, welcome letter, and a take-home manual, along with step-by-step directions for using the website will be rendered manually to the participants. The participants are allowed to communicate with the trial team through email or telephone, sharing their questions and concerns. For assuring the quality of trial, the trial assistant will be trained during several sessions regarding the interaction with the website, the potential questions and answers of the participants, and the intervention process.

### Ethical Considerations

The Research Review Committee as well as the Regional Ethics Committee (approval # 921835) approved the trial. The trial is registered on the Iran Registry of Clinical Trials [30].

The informed written consent will be obtained from the eligible participants, with its content clearly explained to the subjects by the trial assistant. During the project, participants will be given means of communication in order to share their questions and concerns.

The research tools, including questionnaires, will be completely anonymous. However, a unique code will be included in each form in order to manage further references. Access to the DPHR Internet app requires a user name and password, which are provided by the trial assistant. All participants’ information will be securely stored and will only be accessible to authenticated trial team members.

### Intervention Development

The Web-based DPHR app is designed to empower diabetes patients with self-care information and tools. The major aim of this study is to systematically develop an evidence-based Internet app, which is ultimately validated by the local endocrinologists. The app allows participants to easily enter their monitoring data, to view their history of progress, to get informed about their appointment schedule, and to learn about their disease. The patients will also be able to share the blood glucose and lab result trends with their physicians for further advices.

The participants in the intervention arm will receive the Web-based app as well as usual care. On the other hand, the control arm will continue to receive usual care.

The trial team and the software provider will be in charge of the DPHR development, updates, and maintenance. The app interface will be evolved and optimized throughout the trial using heuristic usability evaluation techniques [31] by medical informatics specialists, endocrinologists, and also the participants.

The trial will last for 4 months. The major treatment process delivered by the attending physicians will not have any impact on the intervention and vice versa. During the study, participants may receive extra visits and lab tests in addition to their routine care plan, in order to keep careful track of changes in their health information.

### Sample Size

For estimation of sample size, we did not find any study regarding self-care in Iranian population. However, a study has demonstrated that self-care activities and quality of life are correlated [32]. Therefore, sample size for this study was estimated based on the similar study in the country of Iran [33]. A sample size of 60, corresponding to the formula, has been estimated. Considering confidence interval 95%, a power of 80%, and a dropout rate of 20%, 72 subjects will be required. A total of 36 subjects will be randomly assigned to the intervention arm to receive the DPHR tool, and the remaining 36 subjects assigned to the control to receive the usual care.

### Outcome Measure

The investigators will employ the Summary of Diabetes Self-care Activities Measure-revised for assessing self-care behaviors [34]. A study has reported the validity and reliability of this instrument, with an internal consistency of 0.47 and a correlation of 0.4 [34]. Also, the patient adherence to a planned visit will be measured.

In this study, researchers will examine any potential correlation, rather than cause and effect, between the app usage and the diabetes follow-up clinical indicators. Such indicators which are based on the routine follow-up procedure of diabetes clinics are: fasting blood sugar (FBS); 2-hour postprandial blood sugar; bedtime blood sugar; blood pressure; weight; height; BMI; lipid profile (total cholesterol, triglyceride, HDL, and LDL); and HbA1c.

### Data Collection

One primary outcome and several secondary outcomes will be assessed with several validated assessment tools. Face and content validity of the questionnaire will be assessed through the expert opinions (including two endocrinologists, one health informatics specialist, and one methodologist) and valid literature. Also, the questionnaire will be completed using structured interviews through the person who is not a member of trial team. The subjects will complete the questionnaire on paper.

Each participant will be requested to complete the questionnaire in-person at the reference clinic at the time of the scheduled diabetes follow-up. Statistics on website visits will be obtained using the access logs by the Web server. After the end of the trial period, the participants’ opinions and experiences on the DPHR tool will be assessed using another questionnaire.

### Analysis Plan

All analyses will be 2-tailed and statistical significance is considered as a *P* value of ≤.05. Data will be analyzed using SPSS. χ^2^ test will be used for determining differences in subject groups based on data collected from baseline, and postintervention (significance interval of 95% will be considered). A *t* test will be used to compare the control and intervention groups, while a chi-square test will be used for continuous and categorical variables, respectively. The distribution of variables, website usage statistics, and website user satisfaction are examined using descriptive analysis.

Examination of the impact of the DPHR tool on self-care will be performed using a linear mixed model at the end of the trial period. The longitudinal nature of data collection will result in a repeat in the measure of analysis of variance using data from all trial assessment points. Missing values will be handled using multiple imputation [35]. All analyses will be done according to intention-to-treat principle.

Before inferential analysis, the homogeneity of variances of distributions in the study groups will be confirmed. The sequential logistic regression will be used for adjusting baseline characteristics and possible confounders. Dropouts from the study will be measured by no-use of the app tool for more than one month. Also, participants in the intervention group must enter blood glucose measurements at home (FBS, bedtime, 2 hours after lunch, and 2 hours after dinner) at least four times a week, otherwise, they are eliminated from the trial. The access data will be assessed using the Web server access log.

## Results

The recruitment of participants is still ongoing and preliminary results will be published in early 2016.

## Discussion

### New Insights for the App

The results from this trial will propose new insights on the app of DPHRs for patient involvement and participation with their care process. Initially in this trial, we will identify the most important data elements to consider in the design of a Web-based tool for improvement of self-care activities related to diabetes patients and then we will assess the effect of this systematically developed tool.

These trial findings will be of great help and usefulness to endocrinologists who are currently facing the challenge of timely and rapid treatment decision making in the ambulatory settings. The results are likely to be generalizable to other ambulatory endocrine/diabetes centers.

It is envisaged that results from this trial could improve diabetes patients in terms of self-care activities. In addition, our results might be used as a basis in further similar research aiming at patient empowerment using Web-based tools.

The strengths of our trial design are the evidence-based development process of the DPHR tool (based on a systematic review), the inclusion of local experts’ opinions, and the eventual refinement of the adopted design using the usability techniques. Both the value of evidence-based content development and the importance of usability testing in the app development process have been emphasized in several studies [36-38], pointing to the facts that such considerations have rarely been used across similar works [36].

### Limitations

There are a few limitations in our study; these are listed in the following paragraphs.

The primary need to recruit participants with minimum computer and Internet literacy might be a serious limiting factor. Generally, in Internet-based interventions, issues such as digital divide, computer literacy, age, and interest in technology can be effective in participant recruitment. Usually, the younger people, computer literates, and those with Internet access have the most tendency in participating in such studies. This trial is not an exception in principle. Also, a study has shown that middle-age well-educated women who are reasonably well off financially tend to participate in PHR research [37]. Such tendencies may present bias in our findings, and thus our trial may not necessarily represent the actual distribution of the population being studied.

The trial sample will only represent type 2 diabetes patients. It is possible that the findings will not be generalizable to other types of diabetes disease.

Due to the nature of intervention, the investigators may frequently request the presence of the subjects for the interviews. This may cause discomfort and stress to some of the participants. To address such issues, we will provide financial incentives, such as free visits or free laboratory testing in order to encourage better involvement.

Another potential limitation for this trial is the attrition rate. It is possible to have a high rate of participant dropout and subsequently a significant loss of data. Therefore, reminders via telephone contact, email, and SMS will be used.

## Abbreviations

app: application
BMI: body mass index
DPHR: diabetic personal health record
FBS: fasting blood sugar
HbA1c: glycated hemoglobin
LDL: low-density lipoprotein
PHRs: personal health records
RCT: randomized controlled trial
SMS: short message service
